# Progress of melatonin in the treatment of intervertebral disc degeneration

**DOI:** 10.3389/fphys.2025.1529315

**Published:** 2025-05-14

**Authors:** Jianlin Yin, Lei Wan, Kuaixiang Zhang, Jiangjia Yang, Man Liu, Mingyu Zhao, Jitian Li

**Affiliations:** ^1^ Henan University of Chinese Medicine, Henan Luoyang Orthopedic Hospital (Henan Provincial Orthopedic Hospital), Zhengzhou, China; ^2^ Department of Osteology, The Second Affiliated Hospital of Luohe Medical College, Luohe, China; ^3^ Graduate School, Hunan University of Chinese Medicine, Changsha, China

**Keywords:** intervertebral disc degeneration, melatonin, oxidative stress, low back pain, inflammation, cell death

## Abstract

The most common degenerative condition affecting the musculoskeletal system, and the leading cause of persistent low back pain, is intervertebral disc degeneration (IDD). IDD is increasingly common with age and has a variety of etiologic factors including inflammation, oxidative stress, extracellular matrix (ECM) degradation, and apoptosis that interact with each other to cause IDD. Because it is difficult to determine the exact pathogenesis of IDD, there is a lack of effective therapeutic agents. Melatonin has been intensively studied for its strong anti-inflammatory, antioxidant, and anti-apoptotic properties. Melatonin is a pleiotropic indole-stimulating hormone produced by the pineal gland, which can be used to treat a wide range of degenerative diseases. Therefore, melatonin supplementation may be a viable treatment for IDD. This article reviews the current mechanisms of IDD and the multiple roles regarding melatonin’s anti-inflammatory, antioxidant, anti-apoptotic, and mitigating ECM degradation in IDD, incorporating new current research perspectives, as well as recent studies on drug delivery systems.

## 1 Introduction

One of the main causes of disability and a significant socioeconomic burden is low back pain (LBP), which is acknowledged worldwide. It is well known that 80% of people will experience low back pain at some point in their lives. In the past 20 years, the prevalence of LBP has increased by 50% worldwide, and it is one of the main causes of disability (Violante et al., 2015; Diwan and Melrose, 2023; Hartvigsen et al., 2018). Moreover, one of the main causes of LBP, intervertebral disc degeneration (IDD), accounts for about 40% of all cases of LBP (Cheung et al., 2009). Currently, conservative and surgical approaches are the primary methods used to treat IDD. These two treatment modalities have formed a more mature system and have achieved good results in the clinic (Wu et al., 2020; Xin et al., 2022). Analgesics, anti-inflammatory medications, and physical therapy are the mainstays of conservative treatment, nevertheless, these approaches are only effective in treating symptoms, which merely conceal or postpone the progression of IDD (Xin et al., 2022; Kamali et al., 2021). As a last resort, surgery is used for procedures like spinal fusion or total disc replacement. Modern surgical methods are highly advanced and appropriate for the great majority of individuals with IDD. However, there are dangers associated with surgical treatment as well, such as operative site infection or problems from neighboring segment disease (Xin et al., 2022; Phan et al., 2021; Takahashi et al., 2006). To make up for the limitations of the current clinical therapeutic approaches, a novel therapeutic technique for suppressing IDD must be developed. Small molecules, which are organic substances with a molecular weight of less than 900 Da, have been shown to have regenerative effect on IDD recently. In order to treat discogenic pain and repair damaged intervertebral disks (IVD) by reestablishing tissue homeostasis, several small molecules have demonstrated encouraging outcomes in both *in vivo* and *in vitro* studies (Kamali et al., 2021; Pan et al., 2018; Liu MY. et al., 2022; Krupkova et al., 2014), which provided ideas for follow-up research.

As a small molecule drug, melatonin has been proved to promote osteochondral development, repair (Zhang et al., 2010; Gao et al., 2014). Except that melatonin has anti-inflammatory, antioxidant and anti-apoptosis effects, this is consistent with the direction of IDD treatment research. Melatonin has great potential in treating IDD (Tian et al., 2021; Zhang Y. et al., 2019; He et al., 2018; Chen et al., 2020a). Thus, the purpose of this review was to give a thorough theoretical foundation for future pertinent research while concentrating on the possible therapeutic principles of melatonin in IDD (Table 1).

**TABLE 1:** Effect of melatonin on IDD in different models.

Model	Dosage	Model of administration	Effects	References
mouse NPCs	0.01, 0.1,1 μM	Incubation with melatonin	Melatonin inhibits inflammatory response, oxidative stress, and ferroptosis in NPCs	Dou et al. (2023a)
rat NPCs	0.1,1,10,100 μM	Incubation with melatonin	Melatonin inhibits apoptosis in NPCs	Chen et al. (2019)
rats NPCs	1 mM	Incubation with melatonin	Melatonin inhibits myeloid apoptosis by suppressing autophagy in the PI3K/Akt pathway in a high glucose environment	Li et al. (2021a)
human NPCs	0.1,0.5,1,5,10,50,100 μM	Incubation with melatonin	Melatonin activates the ERK1/2 signaling pathway to improve cell survival and function	Ge et al. (2019)
human NPCs	.5,1,2 μM	Incubation with melatonin	Melatonin inhibits NLRP3 inflammatory vesicle activation and attenuate NPCs degeneration through the EGR1/DDX3X pathway	Zhao et al. (2024)
human NPCs	1 mM	Incubation with melatonin	Melatonin activates autophagy through the NF-κB signaling pathway and alleviates ECM degradation	Chen et al. (2020a)
human NPCs	/	/	Melatonin reverses TNF-α-induced metabolic disorders in human myeloid cells through MTNR1B/Gαi2/YAP signaling	Qiu et al. (2022)
rats	5 mg/kg-MLT, 10 mg/kg	intraperitoneal injection	Melatonin reduces the degree of IDD in rats	Dou et al. (2023b)
rats	0,1,3,5 mg/kg	intradiscal injection	Melatonin attenuates degenerative disc degeneration by down-regulating DLX5 via the TGF/Smad2/3 pathway in NPCs	Zhang et al. (2024)
rats	30 mg/kg/per week	intraperitoneal injection	Melatonin reduces the progression of pain and IDD	Chen et al. (2020b)
rats	50 mg/kg/d	intraperitoneal injection	Melatonin alleviates inflammation and IDD processes in rats	Zhang et al. (2019a)
rats	500 μM	intradiscal injection	Melatonin restores BMAL1 expression and ameliorates the IDD process in a compression-induced rat model	Wang et al. (2022a)
plasma	/	/	Decreased plasma melatonin levels are associated with increased pro-inflammatory cytokines	Tian et al. (2021)
NPMSCs	1 μM	Incubation with melatonin	Melatonin attenuates NPMSCs damage by activating the PI3K/Akt pathway	Huang et al. (2023)
rats AF Cells	1 mM	Incubation with melatonin	Melatonin regulates the ROS/NF-κB pathway to ameliorate the inflammatory environment and mitigate cellular senescence	Li et al. (2021b)
rats EP Cells	0,0.5,1,2,5 μM	Incubation with melatonin	Melatonin reduces oxidative stress-induced apoptosis in EPCs by promoting autophagy	Zhang et al. (2019b)
rats EP Cells	1.10 mM	Incubation with melatonin	Melatonin delays the degeneration of EPCs by inhibiting the NF-κB pathway	Wu et al. (2021)

## 2 Anatomy and mechanisms of IDD

The annulus fibrosus (AF), endplate cartilage (EP), and nucleus pulposus (NP) make up IVD (Zhang et al., 2010; Gao et al., 2014). Nucleus pulposus cells (NPCs) and ECM rich in collagen II and proteoglycans make up NP (Zhang et al., 2010; Gao et al., 2014). The presence of negatively charged side chains in proteoglycans causes the NP to become very hydrated at high osmotic pressures, enabling the IVD to withstand compressive stresses and experience reversible deformation (Zhang et al., 2010; Gao et al., 2014). The passage of fluid and solutes into and out of the disc is intimately linked to the CEP, a layer of hyaline cartilage covering the caudal and cephalad ends of the disc (Zhang et al., 2010; Gao et al., 2014). AF surrounding the outer region of the IVD, is a highly fibrous and well-organized tissue. It consisted of multiple layers of concentric lamellae, angularly laminated in the fiber direction, which restrict the mobility of the IVD and contain the internal NP (Torre et al., 2019). AF is significant in biomechanical characterization of IVDs, because its structural integrity is vital for limiting NP and maintaining physiologic intradiscal stress under load, IDD is the result of degenerative changes in the biomechanical and structural features of the IVD, such as AF cracking and NP volume loss (Chu et al., 2018).

Extracellular matrix (ECM) breakdown, oxidative inflammation, and apoptosis are the most common pathogenic alterations linked to IDD (Clouet et al., 2009; Chen et al., 2024). According to the current study, NPCs (IVD Cells in the central region) keep the ECM in a balanced state. The anabolism and catabolism of ECM are in dynamic balance in a healthy IVD. The maintenance of ECM integrity depends on type I and type II collagens, which give tensile strength, and water-bound proteoglycans, such as aggregated proteoglycans. However, dysregulation of NPC metabolism results in a reduction in their capacity to synthesize ECM components and an increase in the secretion of molecules that promote ECM degradation, such as matrix metalloproteinases (MMP) and metalloproteases with platelet-responsive protein motifs (ADAMTS). IDD typically happens when the ECM’s balance is upset (Vo et al., 2013; Roberts et al., 2000; Liang et al., 2022; Le Maitre et al., 2007).

The inflammatory response also has a significant impact. IVD degeneration is characterized by increased levels of the pro-inflammatory cytokines tumor necrosis factor (TNF), interleukin (IL)-1α, IL-1β, IL-6, and IL-17 secreted by IVD cells, which promote ECM degradation, chemokine production, and IVD cells cellular phenotypic change. Concurrently, chemokines released by degenerative discs encourage immune cell infiltration and activation, so intensifying the inflammatory cascade response (Xin et al., 2022; Risbud and Shapiro, 2014).

This process is also influenced by oxidative stress, which interacts with the inflammatory response. Excess reactive oxygen species (ROS) trigger the nuclear factor kappa-B (NF-κB) and mitogen-activated protein kinase (MAPK) pathways, causing an imbalance in the synthesis and breakdown of ECM in IVD cells as well as an increase in the secretion of pro-inflammatory factors. These alterations eventually cause NPCs to undergo apoptosis and maintain an inflammatory microenvironment, which exacerbates IVD disruption and ROS production (Zhou et al., 2010; Zhu et al., 2019). Cellular senescence is an irreversible cell cycle arrest caused by various external stimuli or telomere unraveling. Senescent cells exhibit a variety of morphological changes as they aggregate into clusters, increase in size, and become flattened and vacuolated. In addition, these cells will also fail to replicate in response to mitotic stimuli and abnormally secrete pro-inflammatory cytokines and matrix-degrading proteases (Acosta et al., 2008). Senescent cells alter the balance of catabolic and anabolic pathways produced by the ECM, a potential cause of IDD (Gruber et al., 2009). Increased ROS in the IDD is linked to NPCs senescence, and it has been demonstrated that raised surface ROS and activated NF-κB cause NPC senescence (Wang J. et al., 2022; Li F. et al., 2021). IVD cells experience senescence, irreversible growth arrest, the synthesis of MMPs and pro-inflammatory cytokines, and overstress, which culminates in programmed cell death, as they age and degenerate (Yurube et al., 2023).

**FIGURE 1:**
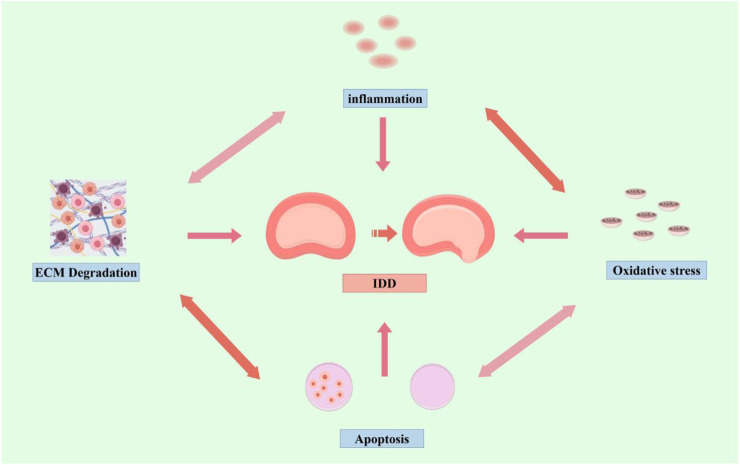
Mechanisms of IDD (by Figdraw). Inflammation, oxidative stress, apoptosis, and ECM degradation, promote the progression of IDD.

In conclusion, although there is no clear standard explanation for the mechanism of IDD, current studies have demonstrated that multiple factors such as ECM degradation, inflammation, oxidative stress, and apoptosis are interconnected and interact with each other, and work together to promote the progression of IDD (Figure 1).

## 3 Pharmacologic effects of melatonin

A naturally occurring hormone, melatonin is mostly secreted by the pineal gland (Wang SY. et al., 2020). Melatonin, also known as N-acetyl-5-methoxytryptamine, is a neurohormone that maintains circadian rhythms (Pévet, 2016). It is well known that melatonin is widely used for its ability to improve circadian rhythms (Cajochen et al., 2003; Vasey et al., 2021; Zisapel, 2018; Hirayama et al., 2023). Meanwhile, melatonin is safe and widely used in various diseases (Vine et al., 2022; Esposito et al., 2019; Reiter et al., 2002) such as cancers, cardiovascular disease, gastrointestinal disorders, mood disorders (Talib, 2018; Bhattacharya et al., 2019; Ahmad et al., 2023; Favero et al., 2018). Moreover, melatonin has been extensively documented to have a role in the prevention of a number of degenerative illnesses, such as Parkinson’s, Alzheimer’s, osteoporosis, osteoarthritis, IDD, and others (Shen et al., 2022; Sumsuzzman et al., 2021; Tamtaji et al., 2020; Li et al., 2019; Hardeland et al., 2015; Zhang et al., 2022; Li et al., 2022).

Melatonin works in a variety of ways. From an anti-inflammatory standpoint, it inhibits inflammation-promoting processes. TNF-α, IL-6, and IL-1β are among the inflammatory factors whose release is inhibited by melatonin (Zhang Y. et al., 2019). Furthermore, it initiates the anti-inflammatory network through SIRT1 activation, nuclear factor erythroid 2 related factor (Nrf2) upregulation and NF-κB downregulation, as well as the production of IL-4 and IL-10, two anti-inflammatory cytokines (Hardeland, 2019; Arioz et al., 2019). In addition, melatonin acts as an antioxidant with powerful antioxidant properties. Unlike certain classical antioxidants, melatonin reacts with free radicals in a cascade and can be generated under mild oxidative stress. These characteristics of melatonin shield organisms from harmful oxidative damage (Tan et al., 2015; Reiter et al., 2018). Apart from its anti-inflammatory and antioxidant properties, research suggests that melatonin may also have anti-apoptotic effects, it had also been shown that melatonin inhibited apoptosis through activation of the Sirt1/Nrf2 pathway (Zhang W. et al., 2023). All in all, the potent anti-inflammatory, antioxidant, and anti-apoptotic properties of melatonin have been acknowledged in a variety of fields.

The safety of exogenous melatonin is also well established; in a 1967 study, mice survived the administration of a dose of 800 mg/kg of melatonin without experiencing any notable side effects, and its lethal dose is currently unknown in clinical practice (Author anonymous, 2004). However, there is no proof of ophthalmotoxicity, hepatotoxicity, nephrotoxicity, or myelotoxicity. In fact, “melatonin has been given to humans at relatively high doses (1 g per day orally) for 1 month” and researchers have noted high plasma concentrations of melatonin, headaches, dizziness, and drowsiness (Schomerus and Korf, 2005). In summary, melatonin has a good safety record and is being studied extensively in the areas of anti-inflammatory, antioxidant, and anti-apoptotic properties. Therefore, melatonin offers potential as a drug for the treatment of IDD because these effects align with the path of research on IDD treatment.

## 4 Melatonin and IDD

### 4.1 Melatonin regulates inflammation

IDD is largely caused by an inflammatory response, and inflammatory mediators are essential to IDD (Li Z. et al., 2023). According to the study, several pro-inflammatory cytokines such as TNF-α, IL-1β, IL-17, IL-1α, and IL-8 are increased significantly in IDD (Li Z. et al., 2023; Li H. et al., 2023; Shao et al., 2021). These cytokines encouraged IVD cell phenotypic alterations and ECM degradation. Furthermore, the deteriorated disc’s production of chemokines encourages immune cell infiltration and activation, which intensifies the inflammatory cascade response. Inflammatory processes exacerbated by cytokines IL-1β and TNF-α are considered to be key mediators of IDD, and therefore, IL-1β and TNF-α are the most important pro-inflammatory cytokines (Wang Y. et al., 2020). According to recent research, the development of IDD is substantially correlated with the activation of NOD-like receptor thermal protein domain associated protein 3 (NLRP3) inflammatory vesicles (Chao-Yang et al., 2021). It has been reported that NLRP3 inflammatory vesicles promote the release of IL-1β, and in addition, IL-1β upregulated the initiation and activation of NLRP3 inflammatory vesicles by enhancing NF-κB signaling and mitochondrial reactive oxygen species (mtROS) production, which constitutes the NLRP3-IL-1β inflammatory loop (Chen et al., 2020b; Chao-Yang et al., 2021; Huang et al., 2021; Fu and Wu, 2023). Melatonin was found by *Chen et al.* to downregulate NF-κB signaling and mtROS generation, which in turn reduced the activation of NLRP3, IL-1β, and prevented the IL-1β-NLRP3 positive feedback loop in NPCs (Chen et al., 2020b). Nicotinamide phosphoribosyl transferase (NAMPT) is an intracellular enzyme with pro-inflammatory properties (Martínez-Morcillo et al., 2021; Audrito et al., 2020). According to previous studies, NAMPT was involved in the process of IDD, it could contribute to the pathogenesis of IDD (Shi et al., 2022; Yi et al., 2023). *Huang et al.* revealed that melatonin inhibits the activity of NLRP3 and NAMPT inflammasomes, which may mitigate the matrix degradation caused by TNF-α. Furthermore, by blocking NLRP3 inflammasome activity via MAPK and NF-κB signaling in NPCs, NAMPT downregulation inhibited TNF-α-induced matrix degradation (Huang et al., 2020). Macrophage (Mφ) was rapidly polarized to M1-type in the inflammatory state, and degenerating IVD cells could also secrete inflammatory mediators to activate Mφ, accompanied by a rise in pro-inflammatory factors (Nakazawa et al., 2018; Yang et al., 2019; Takada et al., 2004). *Dou et al.* showed that lipopolysaccharide (LPS) induced M1-type Mφ polarization with pro-inflammatory properties. Melatonin could suppress M1-type Mφ polarization and alleviate inflammation at the same time. Melatonin inhibited M1-type Mφ polarization and ameliorates inflammation-induced nasopharyngeal carcinoma injury (Dou et al., 2023b). Furthermore, *Wu et al.* discovered that lipopolysaccharide (LPS) stimulation caused damage to EP cartilaginous cells (EPCs). However, melatonin reduced inflammation and ECM degradation of EPCs via the nuclear factor Kappa-B pathway, lessening the detrimental effects of LPS on EP (Wu et al., 2021). Apart from NPCs and EPCs, Li et al. discovered that melatonin significantly decreased the ROS content and NF-κB pathway activity in TNF-α-treated AF cells. Additionally, the protein expression of p16 and p53, which indicate cellular senescence, was also decreased. These findings imply that melatonin inhibits AF cellular senescence by controlling the ROS/NF-κB pathway in an inflammatory setting (Li et al., 2021b). According to a clinical trial by Tian et al., melatonin supplementation raised plasma melatonin, which may have major therapeutic effects by reducing inflammation. Higher plasma melatonin was also linked to lower levels of IL-6 and TNF-α (Tian et al., 2021). Taken together, these effects attenuated inflammatory damage and prevented inflammation-mediated IDD by interfering with the production of pro-inflammatory cytokines, specifically IL-1β and TNF-α, and suppressing M1-type Mφ polarization.

### 4.2 Melatonin relieves oxidative stress

Oxidative stress has a high correlation with inflammation (Reuter et al., 2010). Inflammation results from oxidative stress, which is an imbalance between the generation of ROS and their removal by defense mechanisms. Numerous transcription factors can be activated by oxidative stress, which results in the differential expression of several genes involved in inflammatory pathways (Hussain et al., 2016).

ROS as a major performer of oxidative stress, is a class of unstable, highly reactive molecule (Feng et al., 2017; Nasto et al., 2013). It has been found that the progression of IDD is closely related to ROS accumulation and oxidative stress. Oxidative stress promoted apoptosis, triggered inflammatory response, and exacerbated ECM degradation, thereby exacerbated the IDD process (Wang Y. et al., 2023). Melatonin, a potent antioxidant, can reduce oxidative stress damage (Tan et al., 2015; Morvaridzadeh et al., 2020). ROS levels and malondialdehyde (MDA) as important indicators for assessing oxidative stress (Hu et al., 2021). *He et al.* revealed that melatonin maintains cell viability of NPCs under oxidative stress, leading to decrease in apoptosis rate, ROS levels and MDA, reducing damage from oxidative stress (He et al., 2018). Mesenchymal stem cells (MSCs) have become a novel treatment option in recent years because of their paracrine actions, capacity to develop into intervertebral disc cells, and ability to restore degraded cells (Bhujel et al., 2022; Vadalà et al., 2016). Mesenchymal stem cells from the nucleus pulposus were identified and grown by Risbud et al. (2007), offering a novel strategy for endogenous healing of degenerative disc degeneration. Nucleus pulposus mesenchymal stem cells (NPSC) were present in normal and degenerative NP tissues. NPSCs were better adapted to the hypoxic hypertonic microenvironment of degenerative discs than exogenous stem cells (Huang et al., 2019; Liu et al., 2019). *Huang et al.* revealed that accumulation of ROS in the intervertebral disc would cause to senescence and apoptosis of NPSCs, ultimately leading to irreversible disc degeneration. Melatonin could effectively alleviate oxidative stress-induced excessive apoptosis of NPSCs and mitochondrial dysfunction by activating the PI3K/Akt pathway (Huang et al., 2023). In conclusion, it can be said that melatonin prevented inflammation-mediated IDD by interfering with the production of pro-inflammatory cytokines like TNF-α and IL-1β. Additionally, melatonin is able to lower ROS levels and reduce oxidative stress damage. These findings suggest that melatonin may be a promising treatment for IDD.

### 4.3 Melatonin inhibits IVD cell death

IDD has a very complicated pathophysiology, and one of the main causes of the disease is the death of IVD cells, such as NPCs or EPCs, as a result of the apoptotic pathway being activated (Zhang XB. et al., 2021). Therefore, a viable therapeutic strategy for the therapy of IDD is the suppression of IVD cell death.

According to previous studies, melatonin had several antioxidant and anti-inflammatory effects. In addition to this, melatonin regulated apoptosis and autophagy (Fernández et al., 2015; Zhi et al., 2020; Wang L. et al., 2023). *Chen et al.* found that melatonin reversed the expression of apoptosis-associated proteins, such as cleaved caspase 3, cytochrome c, Bax, and Bcl-2, and prevented tert-butyl hydroperoxide (TBHP)-induced apoptosis in NPCs in a dose-dependent manner. Subsequent investigation revealed that melatonin prevented NPCs from dying by causing and reducing IDD via mitochondrial autophagy, offering a possible treatment for IDD (Chen et al., 2019). Extracellular regulatory protein kinases (ERK) signaling has been linked to apoptosis, senescence, migration, differentiation, and proliferation of cells (Sun et al., 2015). *Ge et al.* found that discovered that ERK1/2 activity was considerably elevated by melatonin above a 1 μM concentration, and furthermore, the addition of an ERK inhibitor nearly entirely reversed the effects of melatonin therapy, which prevented NPC apoptosis by cleaving cystatin-3, reducing Bax expression, and boosting Bcl-2. In addition, melatonin (<5 μM) preserved cell cycle arrest in NP cells while increasing the proportion of S-phase cells and lowering the G0/G1 population (Ge et al., 2019). While *Zhang et al.* was examining the function and mechanism of lncRNA MEG3 in melatonin-mediated NPC action, they discovered that the melatonin-regulated MEG3-miR-15a-5p-PGC-1α/SIRT1 pathway may prevent IL-1β-induced inflammation and NPC apoptosis (Zhang C. et al., 2023).

Previous studies have suggested that diabetes-related hyperglycemia may be one of the risk factors for IDD (Jin et al., 2023; Cannata et al., 2020; Alpantaki et al., 2019). *Li et al.* observed an increased apoptosis rate of NPCs in a high glucose culture environment compared to the normal environment. This increase was mainly characterized by increased expression of apoptosis marker proteins such as cleaved Caspase-3 and cleaved PARP. In addition, melatonin was found to inhibit apoptosis of NPCs in high glucose environment. Notably, the researchers looked at the protein expression of p-Akt to explore the potential involvement of the PI3K/Akt pathway in IDD. The findings revealed that, in comparison to control NPCs, high-sugar culture significantly decreased the protein expression of p-Akt, but that melatonin addition partially promoted the expression of p-Akt protein in NPCs cultured in medium supplemented with high sugar concentrations. Furthermore, the PI3K/Akt pathway inhibitor LY294002 was added to melatonin-treated NPCs to prevent the activation of this signaling pathway. The findings demonstrated a large rise in the mRNA expression of autophagy-related genes (Beclin-1, Atg3, and Atg5) and a significant drop in the mRNA expression of anti-apoptotic genes (Bcl-2). Pro-apoptotic genes (Bax and cystatin-3) showed the lowest level of mRNA expression. In conclusion, melatonin reduced apoptosis in NPCs by blocking excessive autophagy in high glucose cells via the PI3K/Akt pathway (Li et al., 2021a). *Zhang et al.* fond that melatonin therapy decreased the frequency of calcification and apoptosis in EPCs. Notably, melatonin increased autophagy and Sirt1 expression and activity in EPCs. The protective effects of melatonin on apoptosis and calcification were reversed when 3-methyladenine blocked autophagy. Conversely, the Sirt1 inhibitor EX-527 decreased both melatonin-induced autophagy and the protective effects of melatonin on calcification and cell death. This indicated that the Sirt1 autophagy pathway mediated the favorable effects of melatonin on EPCs (Zhang Z. et al., 2019). In conclusion, the above studies suggested that melatonin had protective effect on IVD cells, including NPCs, EPCs. Cysteine asparaginase activation and inflammatory vesicles cause cellular pyroptosis, a form of lytic programmed cell death.

In contrast to apoptosis, cellular pyroptosis is characterized by the rupture of the plasma membrane and the release of inflammatory mediators, which speeds up the ECM’s decomposition. According to recent research, as IDD worsens, NLRP3 inflammatory vesicle-mediated pyroptosis is triggered. Targeting cellular pyroptosis in IDD also revealed the ECM’s amazing remodeling capabilities and anti-inflammatory qualities, indicating that cellular pyroptosis plays a role in the IDD process (Luo et al., 2022). *Zhao et al.* discovered that melatonin protects cells from cellular death and ECM degradation by reducing EGR1-induced overproduction of DDX3X and activation of NLRP3 inflammatory vesicles (Zhao et al., 2024). *Xie et al.* discovered that while melatonin dramatically inhibited the activity of NLRP3 inflammatory vesicles and decreased pain behavior in a rat model of radiculopathy, NLRP3 inflammatory vesicles were raised in both a dorsal root ganglion model and a rat model of radiculopathy (Xie et al., 2021). In conclusion, there is some study potential for melatonin’s ability to prevent cellular pyroptosis.

**FIGURE 2:**
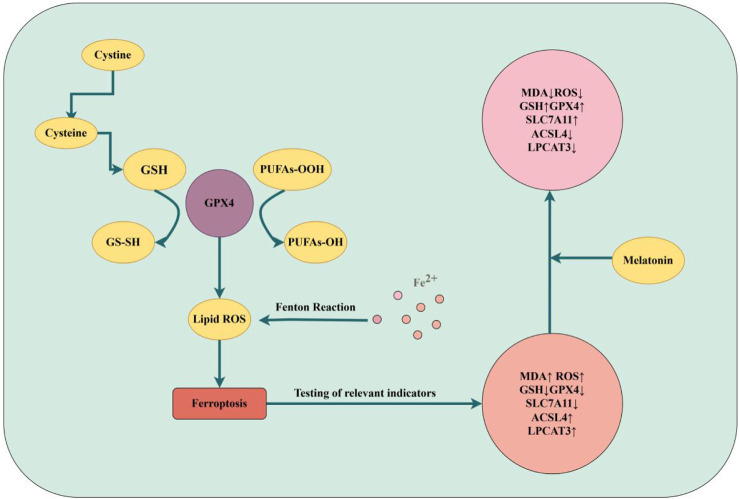
Ferroptosis of IDD (by Figdraw). Mechanisms of iron death and changes in the effects of melatonin on indicators of iron death are described.

According to previous studies, apoptosis and pyroptosis are one of the ways of cell death (Yan et al., 2021; Newton et al., 2024). Apart from apoptosis, ferroptosis has also been linked to IDD (Ohnishi et al., 2022). Ferroptosis is a recently discovered novel form of regulatory cell death (RCD) characterized by iron-dependent mechanisms and accumulation of lipid ROS. Ferroptosis has a high correlation with numerous degenerative illnesses, and its function in the pathogenesis of IDD has drawn more attention (Yan et al., 2021; Fan et al., 2023). In addition, SLC7A11, GPX4, ACSL4, and LPCAT3 are altered during ferroptosis and can be used as marker proteins to detect ferroptosis (Seibt et al., 2019; Chen et al., 2021; Cui et al., 2021; Liu J. et al., 2022). It was shown that in a model of oxidative stress NPCs induced by TBHP, the levels of ferroptosis marker proteins were altered, ferroptosis was observed in this model of NPCs, as evidenced by changes in the expression of PTGS2 and ACSL4 and a decrease in GPX4 and FTH, but the addition of ferroptosis inhibitors reversed these manifestations (Fan et al., 2023; Zhu et al., 2023). This suggested that ferroptosis may be involved in the process of IDD, therefore, studying the relationship between ferroptosis and IDD had become a new research topic. *Dou et al.* revealed that LPS-stimulated macrophages' conditioned medium (CM) produced a ferroptosis-like environment with alterations in ferroptosis marker proteins, such as downregulation of GPX4 and SLC7A11 levels but upregulation of ACSL4 and LPCAT3 levels, which encouraged damage to NPCs. Subsequently, melatonin subsequently attenuated the damage caused by CM to NPCs, in part because it blocked ferroptosis, as evidenced by the overexpression of GPX4 and SLC7A11 levels and the downregulation of ACSL4 and LPCAT3 levels. The ferroptosis-inducing agent erastin further reduced the protective effect of melatonin in NPCs, while ferritin inhibitor-1 (Fer-1) increased it. This finding highlights the role of ferroptosis in the pathophysiology of IDD and raises the possibility that melatonin could be used as a medication for the clinical treatment of IDD. Despite the fact that a number of ferroptosis-related molecules, including GPX4, SLC7A11, ACSL4, and LPCAT3, contribute to the reduction of NPCS injury through the use of melatonin, the study was restricted to cellular experiments and lacked precise and in-depth research on the mechanism of ferroptosis. Additionally, there were no studies involving higher concentrations of melatonin (Dou et al., 2023a). But research on melatonin and ferroptosis is still in its early stages, and many questions remain unanswered. The present research on ferroptosis in melatonin has just touched the surface of the findings; the precise mechanisms, target molecules, and related signaling pathways remain unknown. More research is needed to determine how melatonin, ferroptosis, and IDD are related (Figure 2).

### 4.4 Melatonin regulates ECM remodeling

The ECM’s both catabolism and anabolism are in a state of dynamic equilibrium during normal IVD. Disc degeneration typically happened when multiple stressors upset the balance (Liang et al., 2022; Le Maitre et al., 2007). Collagen II and aggregated proteoglycans are the primary constituents of ECM that preserve its elasticity and volume (Craddock et al., 2018). ECM-degrading enzymes, such as MMP-3, MMP-13, and ADAMTS-4, are upregulated in degenerative IVD specimens in proportion to the degree of degenerative alterations (Vo et al., 2013). Therefore, one of the most important aspects of treating IDD is to keep the ECM stable and avoid its disintegration.

It has been demonstrated that the PI3K/Akt signaling pathway contributes to IDD and that preventing ECM degradation can be achieved by activating this signaling system (Ouyang et al., 2017). By activating the PI3K/Akt pathway, IGF-1 dramatically reduced MMP-13 production and activity in rat endplate chondrocytes, increasing the amount of type II collagen (Zhang et al., 2009). Furthermore, by increasing Akt activity, IGF-1 and bone morphogenetic protein 7 treatment of bovine NP cells synergistically encouraged the buildup of aggregated proteoglycans (Zhang et al., 2009). To sum up, PI3K/Akt pathway activation is critical for reducing ECM degradation. Melatonin also has a role in the regulation of this signaling pathway, as reported by *Li et al.*, melatonin interacts with the membrane receptor MT1/2, regulates the PI3K-Akt signaling pathway, and enhances the expression of Collagen II and aggregated proteoglycans in NPCs, and downregulates the expression of MMP-3 and MMP-9, all of which lead to remodeling of the extracellular matrix (Li et al., 2017). NF-κB pathways have long been recognized as a causative factor in IDD (Zhang GZ. et al., 2021), *Chen et al.* found that melatonin treatment of NPCs for 24 h resulted in a significant increase in the expression of type II collagen and aggregated proteoglycans in NPCs, with a corresponding decrease in the levels of ADAMTS-4, ADAMTS-5 and MMP-3, the underlying mechanism for this discovery, which was also confirmed in animal tests, may be that melatonin reduces ECM degradation by triggering autophagy via the NF-κB signaling pathway (Chen et al., 2020a). Furthermore, by decreasing melatonin receptor-mediated activation of the PI3K/AKT and NF-κB pathways, *Liang et a*l. discovered that melatonin decreased NPCs catabolism and death (Liang et al., 2024).

*Shen et al.* discovered that melatonin facilitated the expression of markers associated with the extracellular matrix, such as COL2A1, ACAN, and SOX9. Meanwhile, cell proliferation in melatonin-treated group increased from day 3 (Shen et al., 2020). *Zhang et al.* discovered that melatonin downregulates matrix MMP-3 levels and increases the expression of collagen II and aggrecan to modify IL-1β-induced ECM remodeling (Zhang Y. et al., 2019). According to certain research, melatonin’s suppression of ECM disruption may be achieved via reducing oxidative stress, inflammation, and apoptosis as well as by influencing autophagy in IVD cells (Hemati et al., 2022).

When considered collectively, these findings imply that melatonin was a medication that preserved ECM homeostasis in IVD and offer fresh perspectives on treating.

## 5 New advances in drug delivery systems

Because of their lack of vascularization, IVD have a limited capacity to heal themselves after injury. Therefore, we need to find a new drug delivery system to better utilize the role of drugs in IVDs. Hydrogel injection, a minimally invasive technique, may represent a novel approach to regenerating or restoring the biological function and structure of IVD. In IVD tissue engineering, hydrogels are widely used due to their high biocompatibility, regulated degradation rates, and non-toxic breakdown products. IVD regeneration is a novel and promising strategy, according to *Hu et al.*, who discovered that implantation of melatonin-containing hydrogels improved *in situ* regeneration of AF tissue and might be utilized to prevent IVD degeneration by maintaining hydration of rat NPs in a rat model of IVD abnormalities (Hu et al., 2023). Even though hydrogel can increase NPCs self-renewal and restore disc height, its poor mechanical qualities could prevent it from being used further (Liu et al., 2021). *Wu et al.* created the composite hydrogel Mel-MBG/SA, an injectable mesoporous bioactive glass/sodium alginate hydrogel loaded with melatonin. This system was validated in rats and offered a hybrid system with sustained melatonin release to reduce inflammation linked to IDD pathology and attenuate oxidative stress caused by IL-1β. successfully reduced rat tail inflammation.This method preserved their injectability and anti-inflammatory benefits while enhancing their mechanical qualities (Wu et al., 2023). In conclusion, melatonin and biomaterials science can be combined to improve drug delivery, but there aren't many studies on the topic. It can be used in a variety of ways in the future, including combining melatonin with hydrogels, melatonin with nanoparticles, and many more. Additionally, as artificial intelligence (AI) advances, it will be possible to use AI to predict drug-material interactions, optimize formulation design, and significantly improve drug delivery through multidisciplinary crossover.

## 6 Conclusions and prospects

IDD is the pathologic basis of degenerative spinal diseases and also one of the main causes of LBP. The pathological changes of IDD include ECM degradation, apoptosis of NPCs, and AF rupture. Mechanistically, IDD is associated with increased MMP in IVD, in addition to oxidative stress, involvement of inflammatory mediators, and activation of apoptotic pathways contribute to IDD. Melatonin is widely used for its ability to improve circadian rhythms and its low toxicity. Notably, it also has potent anti-inflammatory, antioxidant, and anti-apoptotic properties. Many studies have shown that melatonin slows the development of IDD through these pathways.

Notably, ferroptosis, as an emerging research hotspot, has been shown to be involved in the progression of IDD based on existing research evidence. It has been shown that melatonin regulates several iron death-related molecules, such as GPX4, SLC7A11, ACSL4 and LPCAT3, and reduces the damage of NPCs, but the exact and detailed mechanisms and pathways of iron death in this process are not fully understood. There are still several aspects of the specific link between melatonin and ferroptosis that need to be investigated in future studies, first, the sensitivity of NPCs to ferroptosis in the presence of different concentrations of melatonin. Second, the specific mechanisms and pathways between ferroptosis, IDD and melatonin. Third, validation by animal experiments is needed.

Although a large number of studies have shown that melatonin has a role in IDD, its specific clinical application still requires the following work. First, large-scale randomized controlled trials are needed to determine the optimal dose and mode of administration for the prevention or treatment of IDD. Second, detailed studies are needed to report the side effects, resistance to long-term administration and therapeutic effects of melatonin. In addition, the specific mechanism of melatonin’s modulating effect on IDD needs further in-depth study.

**FIGURE 3:**
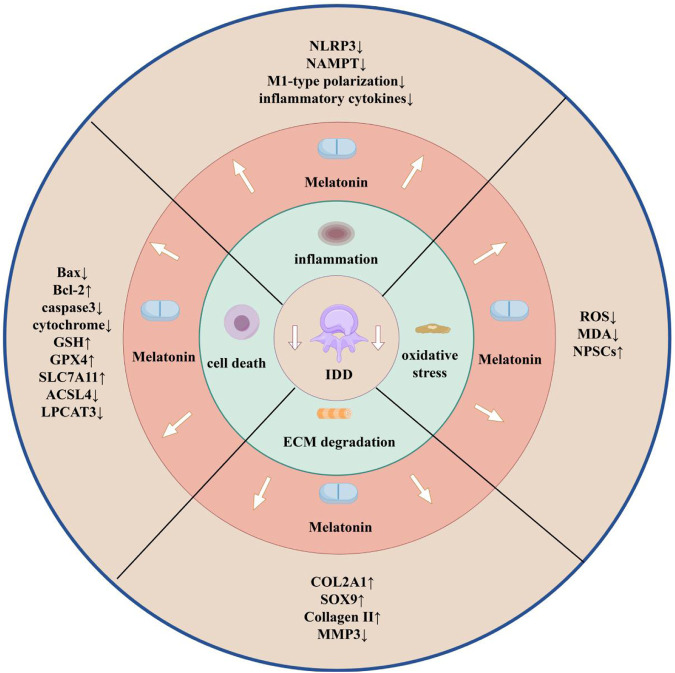
Melatonin and IDD (by Figdraw). Effects of melatonin on IDD through modulation of inflammation, oxidative stress, ECM degradation, and cell death, and changes in each indicator.

In the future research on melatonin and IDD, single-cell sequencing and proteomics technology can be combined to clarify the targeting mechanism of melatonin in different cell subpopulations of the intervertebral disc (e.g., NPCs and annulus fibrosus cells), and in the animal modelling, old animals (e.g., 16-month-old mice) are suitable for long-term melatonin intervention due to the natural degeneration close to the pathological process of human beings; moreover, the absence of vascular properties of the disc limits drug penetration and requires the development of a more penetrating nanocarrier, which can be combined with material science. Moreover, due to the absence of blood vessels in the intervertebral disc, it is necessary to develop nanocarriers with stronger penetration, which can be combined with material science. In conclusion, this review contributes to the understanding of the role of melatonin in IDD, describes the advantages of melatonin that may be used as a potential new treatment for IDD as well as the work needed as a conventional therapy, and helps to stimulate further research on melatonin and IDD (Figure 3).
